# Chemical Composition, Antioxidant Properties and Sensory Aspects of Sponge Cakes Supplemented with Edible Insect Flours

**DOI:** 10.3390/antiox12111912

**Published:** 2023-10-26

**Authors:** Stanisław Kowalski, Dorota Gumul, Joanna Oracz, Justyna Rosicka-Kaczmarek, Anna Mikulec, Barbara Mickowska, Magdalena Skotnicka, Marek Zborowski

**Affiliations:** 1 Department of Carbohydrate Technology and Cereal Processing, Faculty of Food Technology, University of Agriculture in Krakow, 122 Balicka Street, 30-149 Krakow, Poland; rrkowals@cyf-kr.edu.pl; 2 Institute of Food Technology and Analysis, Faculty of Biotechnology and Food Sciences, Lodz University of Technology, 2/22 Stefanowskiego Street, 90-537 Łódź, Poland; joanna.oracz@p.lodz.pl (J.O.); justyna.rosicka-kaczmarek@p.lodz.pl (J.R.-K.); 3 Department of Engineering Sciences, Academy of Applied Science in Nowy Sacz, 1a Zamenhofa Street, 33-300 Nowy Sacz, Poland; amikulec@ans-ns.edu.pl; 4 Department of Plant Product Technology and Nutrition Hygiene, Faculty of Food Technology, University of Agriculture in Krakow, 122 Balicka Street, 30-149 Krakow, Poland; barbara.mickowska@urk.edu.pl; 5 Department of Commodity Science, Faculty of Health Sciences, Medical University of Gdansk, 3a Marii Skłodowskiej-Curie Street, 80-210 Gdansk, Poland; skotnicka@gumed.edu.pl; 6 Department of Health Science, Academy of Applied Science in Nowy Sacz, 2G Kościuszki Street, 33-300 Nowy Sacz, Poland; mzborowski@ans-ns.edu.pl

**Keywords:** edible insects, amino acid profile, nutritional value of protein, texture profile analysis, fatty acid profile, antioxidant properties

## Abstract

The chemical composition, antioxidant properties, and sensory aspects of sponge cakes with the addition of flours from edible insects (buffalo worm, cricket, and mealworm) were evaluated. The addition of edible-insect flours increased the protein, fat, and dietary fiber content in all cases. The utilization of edible insects demonstrated a notable augmentation in the phenolic compounds (especially protocatechuic acid and protocatechuic aldehyde, and syringic, ferulic, and sinapic acids). This resulted in an increase in the antioxidant activity measured against the ABTS radical cation, the DPPH radical, and ferric ions. The antioxidant potential, assessed by four different methods, unequivocally confirmed that the aforementioned polyphenolic compounds found in edible insects provide significant radical-scavenging and antioxidant activity in sponge cakes containing them. The polyunsaturated fatty acid contents were significantly lower in cakes with insect flour compared to the standard wheat cakes. Products and raw materials exhibited high values of the n − 6/n − 3 ratio, which may be associated with negative health effects, with a high oleic acid content. The amino acid score (AAS) for the essential amino acids exceeded 100% for all obtained products. The sponge cakes were accepted by consumers and the taste was the most important predictor for overall acceptability, whereas the structure and appearance had less impact.

## 1. Introduction

An increasing number of institutions are emphasizing the importance of a sustainable food economy. The rising population is exerting greater pressure on ecosystems as a result of heightened demand for food, a fundamental human requirement [1,2]. One of the key issues is the production of sufficient amounts of protein, which is essential for the human body to carry out life processes properly [3]. An alternative to proteins derived from traditional animal and plant production are proteins obtained from edible insects. There is an increasing number of studies on the possibility of using edible-insect preparations in various food products [4,5,6] and their acceptance by consumers [7]. It seems that the sphere of consumer acceptance in particular poses many challenges, especially within the so-called Western culture, where eating insects has clearly negative connotations. However, it is worth emphasizing that an increasing number of studies indicate great potential in this area, especially among young people [2]. It is indicated that products containing edible insects are more widely accepted if they contain the additive in powdered form and not in the form of whole insects (consumer acceptance increases when insects are processed and are not visible in the final product) [7,8]. However, consumer acceptance in Western and developed countries is still limited. Food neophobia, disgust, familiarity, the visibility of insects, and taste seem to be the most significant factors impeding consumers from the consumption of insects as food [8].

Approximately 2300 insect species are edible. However, currently, the European Union (EU) permits only four insect species that comply with the Novell Food Regulations (Reg. 2015/2283) for human consumption. Among them are the larvae of mealworms (*Tenebrio molitor* L.), house crickets (*Acheta domestica* L.), migratory locust (*Locusta migratoria*), and, commencing from 5 March 2023, the larvae of the buffalo worms (*Alphitobius diaperinus* P). The Commission Implementing Regulation (EU) 2017/2470 was released on 30th December 2017. This regulation establishes a European Union list of novel foods in accordance with Regulation (EU) 2015/2283 of the European Parliament and Council on novel foods (OJ L 351, 30 December 2017, p.72). In addition, the European Commission’s Catalogue of Novel Foods provides information on individual component statuses. This catalog contains the names of food ingredients and the information available at the time regarding their status; it is not a definitive guide [9].

In the case of insects such as mealworms or buffalo worms, their larval form is used for food purposes. The dried and ground larvae of these insects contain about 45–49% protein and 14–28% fat [5], and can be used to enrich such products as bread [6], protein bars [10], snack products [11], or as a meat substitute in hamburgers and other meat products [12]. The house cricket (*Acheta domestica* L.) is an insect whose adult form (imago) is used for feed or food. Similar to the previously mentioned insects, it can be widely used for the supplementation of pastry products [6] and the production of pancakes [13].

Edible insects are a good source of protein and fatty acids [5], and also provide such nutrients as minerals (phosphor, potassium, iron) and vitamins, especially ascorbic acid, thiamine, riboflavin, and niacin [5]. All of this means that products with the participation of edible insects can contribute to improving their nutritional status, and in a broader perspective, help fight malnutrition [14].

The aim of this study was to determine the effect of the powders of three species of edible insects (*Tenebrio molitor* L., *Acheta domestica* L., and *Alphitobius diaperinus* P.) on the physicochemical, functional properties, and consumer acceptance of sponge cakes supplemented with the addition of them. The research hypothesis assumes that the addition of the abovementioned edible-insect meals will increase the nutritional value of the obtained products, while maintaining consumer acceptance at an appropriate level.

## 2. Materials and Methods

### 2.1. Materials

Sponge cakes (S) and sponge cakes in which part of the pure refined wheat flour (GoodMills Polska Sp. z o.o., Stradunia, Poland) was replaced with an appropriate insect flour at a ratio of 15% and 30% of the original weight of the wheat flour were used. The following insect flours were used—mealworm, *Tenebrio molitor* (TF) (ZIRP Insects, Wien, Austria); buffalo worm, *Alphitobius diaperinus* (BF) (Isaac Nutrition GmbH, Cologne, Germany); cricket, *Acheta domesticus* (CF) (Sens Food LTD, London, UK). Sponge cakes were obtained under laboratory conditions.

The sponge cakes were made using the cold method with an eggs:sugar:flour ratio of 2:1:1 (2 kg:1 kg:1 kg). The dough was prepared using an Aristan 7 mixer (Kitchen Aid, Orlando, FL, USA), as follows: in the mixing bowl, the egg whites were whipped for 5 min (speed level 10), powdered sugar was added and whipped for 3 min (speed level 8), and the egg yolks were then added and mixed for 1 min (speed level 3). Sieved flour was then added and mixed for 1 min (speed level 2). The dough was formed using a confectionery sleeve into silicone-cake-tin (30 mm diameter) cakes. The cakes were baked in a Miwe roll-in oven (Germany) at 180 °C for 15 min. Cakes were cooled down for half an hour at room temperature and used for further analyses.

### 2.2. Methods

#### 2.2.1. Chemical Analysis

The wheat and edible-insect flour were assessed for the following parameters: ash content (AOAC 923.03) and protein content (AOAC 950.36), with the protein content calculated using an appropriate conversion. In the case of the standard sponge cakes, a conversion factor of 6.25 was used, while in the case of products containing insect flour, conversion factors of 6.25 and 4.76 were used in accordance with the proportions of the individual ingredients in the recipe [15]. The crude fat content (AOAC 935.38), water content (AOAC 925.10), and total, soluble, and insoluble dietary fiber contents (AOAC 991.43) (AOAC, 2006) [16] were also assessed. The analyses were performed in triplicate.

#### 2.2.2. Amino Acid Analysis

Amino acid analysis was performed according to the method of Moore and Stein (1951) [17], Davidson (2003) [18], and Smith (2003) [19]. The samples were freeze-dried and then hydrolyzed using liquid hydrochloric acid (6 M HCl containing 0.5% phenol at 110 °C) for 24 h in an argon atmosphere. The obtained sample was lyophilized again, then dissolved (sodium citrate buffer pH 2.2) and filtered through a 0.45 μm syringe filter before being analyzed using an amino acid analyzer. Ion-exchange chromatography, with a cation ion exchanger and sodium-citrate elution buffer system, followed by postcolumn derivatization with ninhydrin and spectrophotometric detection at 570 and 440 nm, according to the standard protocol of the manufacturer (Ingos, Praha, Czech Republic), were used for the amino acid determination. Sulfur-containing amino acids were analyzed as oxidation products obtained by performic acid oxidation, followed by a standard hydrolysis procedure with HCl. Calibration was done using a standard solution of amino acids (Sigma, St. Louis, MO, USA). The obtained data were assessed using the chromatographic device software CHROMuLAN v 0.91 (Chromulan, Pikron, Ustecky, Czech Republic). As it is destroyed during acid hydrolysis, tryptophan was not determined. Asparagine and glutamine have been designated as aspartic acid and glutamic acid (into which they transform). Analyses were performed in four repetitions.

##### Protein Nutritional Quality

The protein nutritional value was assessed by calculating the amino acid score (AAS) in accordance with FAO/WHO guidelines, based on the quantity and composition of the amino acids [20] (Equation (1)):

(1) $$AAS=\frac{mg\;of\;amino\;acid\;in\;1\;g\;of\;test\;protein}{mg\;of\;amino\;acid\;in\;reference\;pattern\;*}\times 100$$

where * is the recommended amino acid scoring patterns for adolescents and adults, according to the FAO [21] (2013).

#### 2.2.3. Determination of Fatty Acid Profile

The fat fraction was obtained according to the AOAC method 935.38, while the determination of the total fatty acid profile was performed according to the AOAC-approved method 991.39 (AOAC, 2006) [16]. To obtain the methanol esters of the fatty acids, saponification (0.5 N KOH methanol solution) and esterification (12% BF_3_ methanol solution) of the fat sample were performed. The methyl esters were then transferred to the organic phase (hexane). This process was supported by the addition of a saturated NaCl solution. The fat phase was collected into vials and analyzed using a Shimadzu GC2010Plus chromatograph (Shimadzu Corp., Kioto, Japan) with a flame ionization detector (FID). The operating parameters were as follows: FID detector temp. 240 °C; temperature dispenser 240 °C; oven temp. 195 °C to 240 °C (5 °C/min) (240 °C, 10 min). An SH-FAME column (30 m–0.32 mm–0.25 μm) was used, and the carrier gas helium was 1.6 mL/min. The split ratio was 100. Individual fatty acid methyl esters were identified by comparison to the standard mixture of the Supelco 37 component FAME Mix and of CLA isomers (Sigma-Aldrich Co., St. Louis, MO, USA). The analyses were performed in four repetitions.

#### 2.2.4. Color Analysis

The color of the bread crust and crumb was assessed employing the CIELab (L*, a*, b*) system using the Konica Minolta CM-5 spectrophotometer (Konica Minolta Sensing, Osaka, Japan). A measurement angle of 10° and an illuminant of D65 with an 8 mm shutter were used. Ten different locations on the crust and crumb surface were measured to ensure accuracy. The test temperature was maintained at 21 °C throughout. The average color parameters obtained from the measurements were utilized to compute the overall color variation of wheat bread as determined by the formula [22] (Equation (2)):

(2) $$∆E=\sqrt{∆L^{2}+{∆a}^{2}+∆b^{2}}$$

where:

Δ*L* = brightness difference;
Δ*a* = redness difference;
Δ*b* = yellowness difference.

#### 2.2.5. Antioxidant Properties

The extraction procedure: approximately 0.6 g of the sample was shaken in 30 mL of 80 g/100 g ethanol for 120 min (electric shaker type WB22, Memmert, Schwabach, Germany). The obtained extracts were centrifuged (15 min, 4500 rpm; 1050× *g*) (MPW MED, Instruments, Warsaw, Poland). The supernatant was decanted and stored at −20 °C for further analyses.

##### Phenolic Compound Analysis by UHPLC–DAD–ESI–MS/MS

The phenolic compounds in the extracts obtained from the edible-insect flour and sponge cakes were determined using the UHPLC–DAD–ESI–MS/MS method according to the procedure described in detail by Oracz et al. [23].

##### Analysis of Total Polyphenol Content, Flavonoid Content, and Antioxidant Potential by Four Different Methods

The Folin–Ciocalteu reagent method, as described by Singleton et al. [24], was used to measure the total polyphenol content. A 5 mL sample of the extract was diluted to a volume of 50 mL with distilled water in the flask, and 5 mL of the extract was then mixed with 0.25 mL of the Folin–Ciocalteau reagent and 0.5 mL 7% Na_2_CO_3_. The contents were mixed using a vortex (WF2, Janke & Kunkel, Staufen, Germany) and stored for 30 min in the dark. The absorbance was measured at 760 nm using a Helios Gamma 100–240 spectrophotometer (Runcorn, UK). The results were reported in mg catechin/100 g of dry matter of the sample.

The flavonoid content was determined using the spectrophotometric method described by El Hariri et al. [25], using the following method: 0.5 mL of the extract was combined with 0.2 mL 2-aminoethyldiphenylborate reagent with 1.8 mL distilled water into a test tube. The absorbance was measured at a wavelength of 404 nm (Jenway spectrophotometer 6405 UV/Vis, Staffordshire, UK) and the flavonoid content was then expressed in mg rutin/100 g dry matter of the samples.

##### Free-Radical-Scavenging Activity by DPPH

The free-radical-scavenging activity of the samples was measured using the 2,2-diphenyl-1-picrylhydrazyl (DPPH) according to the method of Sánchéz-Moreno et al. [26]. The extract (0.4 mL) was combined with 3.6 mL of the DPPH solution (0.025 g DPPH in 100 mL methanol). The absorbance of the reaction mixture was determined using a Jenway spectrophotometer (6405 UV/Vis, England) at 515 nm within 6 min after adding the DPPH radical to the extract. Trolox (6-hydroxy-2,5,7,8-tetramethylchroman-2-carboxylic acid) was used as the standard, and the results were expressed in mg/g Trolox equivalents.

##### Antiradical Activity by ABTS

Antiradical activity, using the synthetic radical cation ABTS, was determined according to Re et al. [27]. The radical-scavenging activity was estimated by the ABTS (2,2, azinobis (3-ethylbenzthiazoline-6-sulfuric acid)) radical-cation discoloration assay. The ABTS^+•^ stock solution was diluted with PBS buffer to obtain an Abs of 0.700 ± 0.05. Volumes of 2.00 mL of the ABTS^+•^ were added to the diluted sample extracts. The ABTS^+•^ bleaching was monitored at 30 °C and the discoloration after 6 min was used as the measure of antiradical activity. The bleaching rate of the ABTS^+•^ in the presence of the sample was monitored at 734 nm using a Helios Gamma 100–240 spectrophotometer (Runcorn, UK). The results were expressed in mg/g Trolox equivalents 

##### Ferric-Reducing Antioxidant Power (FRAP)

The Oyaizu method [28] was used to evaluate the reducing power of the samples against potassium ferricyanide. An amount of 1 mL of the extract was combined with 5 mL PBS (phosphate buffer with a pH of 6.6) and 5 mL of 1% potassium ferricyanide in the test tube. The mixture was stirred thoroughly and heated in a water bath for 20 min at 50 °C. After cooling, 5 mL of 10% trichloroacetic acid was then added. An amount of 5 mL of the mixture was pipetted into the test tube and combined with 5 mL of distilled water and 1 mL of the 0.1% ferric chloride solution. The absorbance at 700 nm was measured using a Jenway spectrophotometer (6405 UV/VIS, England). The results were expressed in mg/g Trolox equivalents.

##### Determination of Ferrous Ion Chelating Activity 

The chelation of Fe(II) ions by the ethanol extracts was determined as described by Gu et al. [29]. An amount of 1 mL of the extract or blank was combined with 1.85 mL of high-purity deionized water and 50 µL of 2.0 mM FeCl_2_. The solution was mixed for 30 s, and then 100 µL 5 mM ferrozine was added. The reaction mixture was mixed and left to stand at 23 °C for 15 min. The absorbance of the solution was estimated by the spectrophotometric method at 562 nm with a Helios Gamma 100–240 spectrophotometer (Runcorn, UK). A calibration curve was constructed using disodium ethylenediaminetetraacetate dihydrate (EDTA). The results were expressed as mg EDTA/g.

#### 2.2.6. Analysis of Volatile Compounds Using an Electronic Nose

The analysis of volatile flavor compounds using an electronic nose (e-nose) was conducted following the method described by Kowalski et al. [9].

#### 2.2.7. Analysis of Chemical Compounds Using an Electronic Tongue

Instrumental taste analysis of the tested samples was carried out using the Alpha MOS ASTREE II electronic tongue (e-tongue) from Alpha MOS, Toulouse, France, according to the method described by Kowalski et al. [9]. The e-tongue system included a 48-position autosampler, a liquid sensor array (set #5) consisting of seven sensors (SRS, GPS, STS, SPS, UMS, SWS, and BRS), and a reference electrode (Ag/AgCl). The sensor set #5, designed for food and beverage applications, was employed based on [30]. The analysis of each water extract was performed five times. The taste screening analysis was carried out using AlphaSoft software version 14.2 from Alpha MOS (Toulouse, France). This test was used to rank the samples based on taste attributes using a scale from 0 to 12 for intensity. Principal component analysis (PCA) was conducted to differentiate the response signals from the seven sensors.

#### 2.2.8. Differential Scanning Calorimetry

The thermal properties of the sponge cakes were determined by differential scanning calorimetry (DSC) [31]. For this purpose, a MICRO DSC III differential scanning calorimeter from Setaram Instrumentation (Caluire, France) was used. Sponge cake samples (approximately 40 mg) placed in a high-pressure stainless-steel ‘batch’ cell were heated from 15 to 200 °C at 3 °C min^−1^ and then cooled to 15 °C. The analysis was performed in triplicate. The onset (To), peak (Tp), and conclusion (Tc) temperatures, depolymerization temperature range of the RS starch ΔT = (Tc − To), and enthalpy change (ΔH), expressed in J g^−1^ sample, were calculated from thermograms.

#### 2.2.9. Consumer Acceptance Analysis

##### Acceptability of Insect-Based Sponge Cake

The sponge cakes were offered to participants right after they were baked. The study comprised 64 volunteers aged between 19 and 23 years, chosen from the Medical University of Gdańsk’s database of willing participants. Only those without food neophobia were included in the study. Each sponge cake variety, including the control sample, was tested for acceptability on alternate days. The study was conducted as a double-blind study. Each probant received one biscuit with an assessment card, which was appropriately coded. Each participant received a unique code to facilitate the compilation of data from all days. The subjects knew that they were sponge cakes with the addition of insect flour, while the amount of the additive and the type of edible insect were not known to the probants or the staff who conducted the study. The respondents were healthy, did not take any medications or supplements, did not follow any special diets, and did not smoke cigarettes. All participants provided informed consent to take part in the study, which was approved by the Independent Institutional Research Ethics Committee at the Medical University of Gdańsk (NKBBN/346/2021). Prior to the study, an assessment of food neophobia was conducted on all subjects using the Food Neophobia Scale (FNS) [32]. Appearance, aroma, taste, structure, and overall acceptance were evaluated using 7-point visual scale with extreme indicators of “I dislike it entirely” and “I fully appreciate it” [33].

#### 2.2.10. Statistical Analysis

Statistica 13.0, developed by Tibco Software in Palo Alto, was utilized to conduct the statistical analysis. Sponge cake features were tested through a two-way ANOVA on the flour type, percentage of substitution, and their interaction, with a significance level of *p* ≤ 0.05. In cases where the Levene test indicated significant differences, a post hoc least-significant-difference (LSD) Fisher’s test was conducted. The mean ± standard deviation was used to present the results. During the planning phase of the experiment (on the acceptability of the sponge cakes made with insects), an investigation was conducted into the sample size selection at a level that could produce a statistical conclusion with adequate accuracy and confidence. The probability of detecting the effects of a specific size through test power analysis and interval estimation was also explored. The parameters of multiple regression were estimated using the REGLINP command in an Excel 2010 PL spreadsheet considering the concept of shared variability.

## 3. Results and Discussion

### 3.1. Nutrient Composition

The chemical composition of food determines its nutritional value, which affects its usefulness in human nutrition. Both the raw materials and individual sponge cakes differed in the content of specific nutrients (Table 1). Insect flours were characterized by a significantly higher content of all the analyzed ingredients compared to the wheat flour. Among the insect flours, cricket had the highest content of protein (50.68%), lipids (29.01%), and dietary fiber (soluble, 0.24; chitin, 9.92; insoluble, 18.48; total, 18.72%). This resulted in an increase in the content of these compounds in the sponge cakes with insect flours compared to the control sample (Table 1). In other studies, an increase in the content of individual nutrients as a result of supplementation with flour from edible insects in products such as gluten-free bread, pancakes, muffins, or cupcakes was also observed [12,13,34].

### 3.2. Amino Acid Profile and Protein Nutritional Quality

Insect flours were characterized by a significantly higher content of all essential amino acids compared to the wheat flour (Table 2). Sponge cakes made with insect flours also showed a significantly higher content of most essential amino acids compared to the standard product (Table 2).

**Table 2:** Essential amino acid content of the raw materials and sponge cakes (mg/g of protein).

Samples	Essential Amino Acids (mg/g of Protein)								Total EAA
	His	Ile	Leu	Lys	Met	Phe	Thr	Val	
WF	22.02 ± 0.16 ^f^	32.94 ± 0.50 ^d^	63.89 ± 1.85 ^e^	21.33 ± 0.57 ^e^	24.10 ± 3.96 ^de^	46.39 ± 1.46 ^c^	25.67 ± 0.70 ^e^	38.75 ± 1.17 ^f^	275.09 ± 10.09 ^g^
BF	44.76 ± 0.13 ^a^	53.75 ± 1.25 ^a^	82.94 ± 2.01 ^ba^	81.15 ± 2.36 ^a^	24.63 ± 2.98 ^dc^	54.08 ± 1.87 ^a^	49.89 ± 1.12 ^a^	71.20 ± 1.39 ^b^	462.40 ± 8.78 ^a^
CF	28.15 ± 0.18 ^c^	48.12 ± 0.29 ^b^	86.17 ± 0.63 ^a^	62.34 ± 0.70 ^c^	21.44 ± 0.93 ^e^	40.40 ± 0.34 ^e^	44.68 ± 0.46 ^b^	68.20 ± 0.83 ^c^	399.52 ± 2.58 ^c^
TF	39.15 ± 0.51 ^b^	52.18 ± 0.86 ^a^	85.31 ± 1.41 ^a^	67.01 ± 1.08 ^b^	22.18 ± 2.29 ^e^	40.60 ± 0.62 ^e^	48.33 ± 0.95 ^a^	74.46 ± 1.25 ^a^	429.22 ± 4.47 ^b^
S	22.68 ± 2.17 ^fe^	45.82 ± 4.33 ^b^	79.18 ± 3.76 ^ba^	49.69 ± 5.58 ^d^	29.17 ± 1.29 ^a^	51.92 ± 4.67 ^a^	41.07 ± 3.68 ^cd^	55.85 ± 5.02 ^de^	375.37 ± 31.85 ^d^
BF15	23.00 ± 0.25 ^e^	43.09 ± 0.27 ^cb^	72.34 ± 0.61 ^c^	50.49 ± 1.99 ^d^	28.26 ± 0.80 ^ab^	47.14 ± 0.39 ^cb^	38.78 ± 0.23 ^d^	53.09 ± 0.24 ^de^	356.20 ± 2.93 ^e^
BF30	23.98 ± 0.48 ^de^	41.36 ± 0.64 ^c^	68.80 ± 1.15 ^d^	50.15 ± 1.12 ^d^	25.83 ± 0.45 ^cd^	45.06 ± 1.81 ^d^	38.21 ± 0.39 ^d^	52.21 ± 0.44 ^e^	345.60 ± 5.29 ^f^
CF15	21.69 ± 0.32 ^f^	43.11 ± 0.26 ^cb^	75.25 ± 0.35 ^cb^	49.50 ± 1.18 ^d^	28.02 ± 0.21 ^ab^	46.87 ± 1.69 ^cb^	39.64 ± 0.19 ^cd^	54.81 ± 0.46 ^ed^	358.87 ± 2.99 ^e^
CF30	21.45 ± 0.29 ^f^	42.19 ± 0.87 ^c^	72.96 ± 1.33 ^cd^	48.07 ± 0.94 ^d^	27.08 ± 1.09 ^ba^	43.48 ± 0.90 ^e^	39.41 ± 0.41 ^cd^	54.67 ± 1.20 ^ed^	349.32 ± 6.30 ^f^
TF15	24.54 ± 1.29 ^d^	45.49 ± 2.17 ^b^	78.89 ± 3.81 ^ba^	49.48 ± 3.56 ^d^	27.50 ± 0.83 ^ba^	48.66 ± 2.34 ^b^	41.69 ± 2.06 ^c^	58.14 ± 2.08 ^d^	374.37 ± 16.61 ^de^
TF30	23.41 ± 1.68 ^de^	42.01 ± 2.68 ^c^	72.14 ± 4.74 ^cd^	48.76 ± 3.65 ^d^	27.03 ± 0.62 ^ba^	42.53 ± 2.64 ^e^	39.61 ± 2.09 ^cd^	54.44 ± 3.22 ^de^	349.95 ± 20.93 ^fe^

Explanatory notes: meaning of the symbols as in Table 1. Values in the same column marked with different letters are statistically significantly different at *p* < 0.05 ± SD. EAA—essential amino acids. For all sponge cakes made with insect flour, the amino acid score (AAS) surpassed 100% (Table 3). This proves the increased nutritional value of the products obtained.

**Table 3:** Nutritional value of the protein of raw material and enriched products.

Sample	AAS (%)							
	His	Ile	Leu	Lys	Thr	Val	AAA	SAA
WF	137.65	109.79	104.73	44.43	102.69	96.89	171.88	200.91
BF	279.76	179.15	135.97	169.06	199.56	178.00	375.63	162.04
CF	175.94	160.39	141.26	129.88	178.73	170.51	254.22	143.92
TF	244.68	173.93	139.85	139.60	193.33	186.16	299.37	148.99
S	141.75	152.73	129.81	103.52	164.28	139.62	199.16	222.33
BF15	143.76	143.65	118.59	105.20	155.14	132.72	206.40	206.77
BF30	149.86	137.86	112.79	104.48	152.86	130.52	214.13	189.36
CF15	135.59	143.67	123.35	103.12	158.53	137.03	201.47	206.55
CF30	134.09	140.65	119.60	100.15	157.63	136.67	195.57	198.21
TF15	153.34	151.64	129.32	103.07	166.75	145.35	216.43	204.78
TF30	146.33	140.04	118.27	101.59	158.45	136.10	205.48	199.27

Explanatory notes: meaning of the symbols as in Table 1. AAA—aromatic amino acids; SAA—sulfur amino acids.

### 3.3. Fatty Acid Profile

In the fatty acid profile, both insect flours and sponge cakes with their inclusion were characterized by the dominance of monounsaturated fatty acids (MUFAs), ranging from 38.78 in CF30 to 47.97 g/100 g in TF15, and saturated fatty acids (SFAs), ranging from 34.38 for BF30 to 38.27 g/100 g for CF30. The addition of insect flour as an animal-derived ingredient contributed to a reduction in the nutritional value of the final product. Except for the BF15, TF15, and TF30 sponge cakes, the MUFA content in the sponge cakes was significantly lower compared to the standard. Furthermore, the PUFA content and the ratio of total PUFAs to total SFAs were significantly lower in the sponge cakes with the insect flour compared to the standard, except for the BF30 sponge cakes. The use of insect flours also led to a significant increase in the n6/n3 ratio (Table 4). The recommended n-6/n-3 ratio should range from 1:1 to 5:1, while a ratio of 10:1 or higher is associated with negative health effects. A low PUFA/SFA ratio in diets is considered a risk factor for increased blood cholesterol levels. In this study, both the raw materials and the sponge cakes exhibited high values of the n-6/n-3 ratio, indicating an imbalance in the lipid fractions (Table 4).

However, evaluating the nutritional value of insect lipids based solely on fat indexes and the ratio of individual fractions is challenging, as certain fatty acids, like oleic acid, found in higher amounts in sponge cakes with edible-insect flour, have been shown to have beneficial effects on insulin sensitivity [35]. Oleic acid not only prevents insulin resistance but also inhibits endoplasmic reticulum stress, exhibits anti-inflammatory effects, prevents the attenuation of the insulin-signaling pathway, and enhances β cell survival [35]. Studies by Perona et al. (2004) have demonstrated the effectiveness of oils rich in oleic acid in reducing blood pressure and LDL cholesterol levels [36].

### 3.4. Color Analysis

Color is one of the attributes of bakery products, such as cakes, that can influence consumer acceptance. The addition of insect flour has contributed to significant changes in the CIELab parameters and total color change compared to the standard sponge cakes (Table 5). Insect flour has led to a decrease in lightness (L*) as well as a reduction in the levels of red (a*) and yellow (b*) color components. Similar effects on color-parameter values were observed by Pauter et al. [34], who used cricket flour in muffin production. On the other hand, Kowalski et al. [6] observed an increase in the levels of red and yellow color components and total color change in sponge cakes containing mealworms. In that case, chicken eggs were substituted with a plant-based replacement, which may have contributed to the different content of individual color components [6].

### 3.5. Antioxidant Composition and Properties

The characteristics of the raw materials used (insect flours) were presented in a previous publication by Gumul et al. [10]. The utilization of ground edible insects at varying levels between 15% and 30% demonstrated a notable augmentation in the overall polyphenol content of sponge cakes, ranging from 89% to 258%, compared to the standard. Similarly, there was a considerable rise in flavonoid levels, ranging from 3% to 150%, in comparison to the control sample. It is worth emphasizing that the increase in total polyphenols exhibited a direct correlation with the quantity of edible insects employed, with crickets displaying the highest increase and mealworms displaying the lowest. These findings align with the edible-insect characteristics elucidated in the study conducted by Gumul et al. [10], in which cricket flour exhibited the highest polyphenol content, followed by buffalo worm flour and mealworm flour with comparatively lower amounts. By incorporating a substantial proportion of edible insects, wheat sponge cakes displayed an elevation of flavonoid content by up to 2.5 times, particularly when utilizing a 30% inclusion of mealworm in comparison to the control. However, other edible insects did not yield similarly remarkable outcomes (Table 6). In the case of sponge cakes, a 15% inclusion of buffalo and cricket flours resulted in a comparable flavonoid content to that of the control sample. Nonetheless, a 30% inclusion of the flour of these edible insects exhibited an approximately 40% increase in flavonoids relative to the control. The highest flavonoid quantities were observed in sponge cakes incorporating the mealworm flour, particularly at the 30% inclusion level.

Control samples and sponge cakes with the 15% and 30% inclusion of ground edible insects were subjected to HPLC analysis. In the control sample, the presence of phenolic acid derivatives, which may have come from wheat flour used for baking, such as ferulic acid, caffeic acid, *p*-coumaric acid (hydroxycinnamic acids), and protocatechuic aldehyde (hydroxybenzoic acids), were detected (Table 7). These findings are consistent with the results from other studies that have reported wheat flour as a source of the aforementioned acids [37]. Substituting this flour with edible-insect flour, which is characterized by a low amount of hydroxycinnamic acid derivatives [10], resulted in a decrease in their content in wheat sponge cakes containing edible-insect powder (Table 7). In contrast, hydroxybenzoic acids were predominant in sponge cakes with edible insects due to the fact that edible insects themselves contain significant amounts of these acids [10].

The presence of hydroxybenzoic acids in the sponge cakes with edible-insect flour can be influenced by multiple factors. It may partially result from the thermal breakdown of quercetin derivatives, especially rutinoside quercetin, which generates phenolic acids [38]. Considering the presence of quercetin derivatives in edible insects (except for buffalo worms) [10], their thermal degradation may contribute to the increase in the phenolic acid content in sponge cakes. A clear example is the presence of protocatechuic acid in sponge cakes with crickets and mealworms, which was not found in these particular edible insects but appears in the sponge cakes due to the thermal degradation of quercetin derivatives during baking (Table 7).

When analyzing the phenolic acids in sponge cakes with the addition of edible insects, it can be observed that there is a decrease in these compounds (Table 7) due to the thermal decarboxylation of these compounds, including the formation of 4-vinyl guaiacol, during the baking process [39]. Although some of these compounds may originate from the thermal breakdown of quercetin derivatives, or manifest at different stages of sponge cake production, their overall content decreases during baking.

Considering the quantities of hydroxybenzoic and hydroxycinnamic acids, it is evident that the amount of hydroxybenzoic acid is significantly higher than that of the hydroxycinnamic acids in sponge cakes with edible-insect flour. Moreover, a higher inclusion of edible insects results in a greater quantity of these phenolic acids (Table 7). This can be attributed to the dominant presence of hydroxybenzoic acids in edible insects [10]. In summary, it can be stated that, although the baking process leads to losses (up to 66%) of certain phenolic compounds [40], mainly due to the thermal, enzymatic, oxidative degradation, and decarboxylation of phenolic acids [40], it is suggested that the increase in phenolic compounds (primarily phenolic acids) in sponge cakes with edible insects compared to the control is attributed to the addition of these ingredients. It should also be noted that flavonols are completely degraded during baking in sponge cakes; hence, their absence in the product containing edible insects (Table 7). Additionally, the losses of phenolic compounds may be caused by the formation of complexes with polysaccharides [41].

The antioxidant potential, as assessed by four different methods, unequivocally confirmed that the aforementioned polyphenolic compounds found in edible insects provide significant radical-scavenging and antioxidant activity in sponge cakes containing them. Moreover, it was observed that the antioxidant potential, determined by the four methods, was significantly lower in sponge cakes with the 15% inclusion of edible insects compared to those with the 30% inclusion (Table 8). Among all the samples analyzed, sponge cakes with the 30% inclusion of cricket flour exhibited the highest in vitro antioxidant potential (Table 8). This observation can be attributed to the notable presence of hydroxycinnamic acids, such as ferulic acid, caffeic acid, and sinapic acid (Table 7), which have been reported to exhibit superior efficiency in scavenging free radicals [42].

A strong positive correlation was observed between the total polyphenol content (TPC) in the analyzed sponge cakes and ABTS (R^2^ = 0.924), TPC, and DPPH (R^2^ = 0.939), as well as between TPC and FRAP (R^2^ = 0.925), and TPC and iron reduction (R^2^ = −0.859). Furthermore, it can be suggested that, in addition to polyphenols, newly formed compounds during the baking process, such as Maillard-reaction products, may also contribute partially to the high radical-scavenging and antioxidant activity observed [43]. These compounds are likely to significantly influence the radical-scavenging and antioxidant activity of the sponge cakes due to their increased exposure to temperature and the presence of Maillard-reaction substrates, such as sugars, in the sponge cake recipe (Table 8).

### 3.6. Analysis of Volatile Compounds Using the Electronic Nose

An electronic nose was utilized to analyze the volatile compounds present in the control and insect-flour-enriched sponge cakes. Technical abbreviations have been explained upon their initial use. The results are shown in Table 9, revealing thirty-four distinguishable volatile compounds, including aldehydes, ketones, alcohols, esters, pyrazines, lactones, phenols, acids, and pyranones, present in both the control and insect-flour-enriched sponge cakes.

The main flavor and fragrance compounds in both the control and insect-flour-enriched samples were 3-methylbutanal, 2,3-pentanedione, butanal, and 2-propanol, which generate the aromas of malt, sweetness, butter, and a sharp, musty scent, respectively [44]. Nevertheless, it was also observed that the share of different insect flours contributed to a notable change in the concentrations of volatile compounds. The sponge cakes fortified with 30% of cricket and mealworm flours had significantly higher contents of furfural, heptanal, hexanal, 2-propanol, butanoic acid, eugenol, guaiol, and trimethyl pyrazine, but significantly lower contents of acetaldehyde, 3-methylbutanal, 2,3-pentanedione, 2,5-dimethylpyrazine, 2,3-dimethylpyrazine, and 2-phenylethanol compared to the control and buffalo worm-flour-substituted sponge cakes. The presence of aldehydes, ketones, and alcohols in cakes may be due to thermal reactions during baking, such as nonenzymatic Maillard reactions and sugar caramelization. Similarly, these compounds can also be formed by lipid oxidation [45]. The pyrazines are also known to be produced by Maillard and Strecker reactions, and a high amount of protein in the insect flours may intensify the formation of those compounds. These findings indicate that the substitution of wheat flour with different insect flours can influence the aroma profile of baked products. However, the flavor of the final product was greatly influenced by the type of insect flour used, as well as the proportion of each flour in the recipe. For instance, the increased amount of hexanal with the increasing substitution of mealworm flour could be explained by the high fat content in this flour compared to buffalo worm and cricket flours, which could lead to the increased formation of compounds generated by lipid oxidation during thermal processing [44,45]. Moreover, fascinatingly, furfural, a compound formed via the 1,2-enolisation pathway through 3-deoxyosone, was present in significant quantities in the mealworm- and cricket-flour-enriched sponge cakes compared to the other samples. However, almost all of the enriched sponge cakes exhibited high levels of desirable volatile compounds, including 3-methylbutanal, 2,3-pentanedione, and butanal, which provide malt and sweet notes.

### 3.7. Electronic Tongue Analysis 

**Figure 1:**
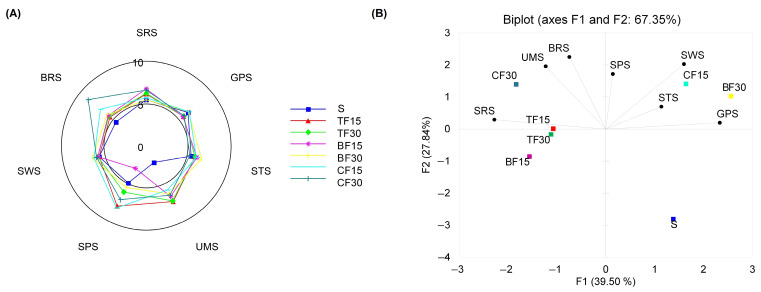
Radar chart representing taste scores of sponge cakes (**A**) and PCA graph showing the relationship among samples and tastes (**B**). SRS (sourness), GPS (metallic), STS (saltiness), SPS (spiciness), UMS (umami), SWS (sweetness), and BRS (bitterness).

The sensory attributes of water extracts from the control sponge cakes and those substituted with edible-insect flour were assessed for taste quality and intensity using an electronic tongue equipped with seven cross-selectivity sensors. The results showed that the substitution of wheat flour with insect flour significantly affected the sensory properties of the samples studied (Figure 1A). Sponge cakes made with insect flours were found to have significantly higher bitter and umami taste, irrespective of the kind and amount of the insect flour used. Furthermore, the addition of insect flour led to a slight increase in the intensities of sour, sweet, and salty tastes in almost all the substituted sponge cakes in comparison to the control samples. With the increase in the quantity of insect flour added, the intensities of these five basic tastes also increased. The bitterness and sweetness of the sponge cakes increased the most with a 30% proportion of cricket flour and buffalo worm flour, respectively. Sponge cakes with 15% and 30% buffalo worm flour had the highest scores for the sour and salty taste. The most prominent change in the intensity of umami taste was noted in sponge cakes supplemented with mealworm flour, irrespective of the share size. Principal component analysis (PCA) was conducted to visualize the relationship between the samples and variables (taste scores) in order to establish any differences and similarities. Figure 1B demonstrates that PC1 and PC2 encompass 67.35% of the entire variance in the data. Of these two principal components, PC1 accounted for 39.50% of the total variation and PC2 explained 27.84% of the variation.

The biplot shows that the tested sponge cake samples and their tastes fall into three distinct clusters. The initial cluster consisted exclusively of control samples, exhibiting less variability in the taste profile and the lowest intensity of the umami taste. The second cluster comprised sponge cakes made with the 15% cricket flour and 30% buffalo worm flour. These samples were notably sweeter and saltier than the other sponge cakes. The remaining sponge cakes that were fortified with insect flours (TF15, TF30, BF15, CF30) were grouped into the third cluster due to their noticeable deviation in relation to the other examined samples, particularly with regard to their umami and bitter taste. The correlation between the increase in umami, bitterness, sourness, saltiness, and sweetness, and the substitution of wheat flour with insect flour, may be associated with the relatively higher protein, mineral, and organic acid content in the insect flours as compared to the wheat flour. Consequently, the findings of this study suggest that the composition and quantity of ingredients used in the production of sponge cakes could influence their sensory properties.

### 3.8. Differential Scanning Calorimetry

Based on the obtained results of thermal analysis of the tested products, it was noted that a single peak was visible in the obtained endotherms in the temperature range from approximately 45 °C to 160 °C, indicating the presence of resistant starch (RS) in those products (Table 10).

Depending on the type of flour used, and its proportion in the recipe, it was found that the amount of RS formed (expressed as enthalpy value) in the sponge cakes ranged from an average of 18% to 53% (i.e., 1.2 to 1.5 times) higher compared to its content in the control sample without insect flour. A significant impact of the type of insect flour used on the amount of RS formed was demonstrated. The highest amount of RS was present in sponge cakes with the mealworm flour, followed by the buffalo worm and cricket flours, regardless of the concentration of these flours. The obtained ∆T values indicate that the RS in the sponge cakes produced with insect flours has a less-crystalline structure compared to the ∆T value obtained for the control sample, suggesting a different influence of the present components in the insect flour, such as proteins and fats, on the retrogradation process of starch. It is known that the interaction between starch and certain nonstarch components, including proteins and lipids, may affect the formation of RS, which exhibits health-promoting properties. It has been observed that protein, as the main food component, plays a significant role in reducing starch digestibility, similar to other components of the food matrix, such as hydrocolloids and phenolic compounds, partly by forming complex compounds with starch [46].

### 3.9. Acceptability of Insect-Based Sponge Cake

The ANOVA results indicate that the quantity of the additive had a significant impact on several sensory assessment factors, whereas the type of additive did not. Only the structure of the sponge cake was affected by the type and quantity of the additive. It is worth noting that the assessed attributes were mainly influenced by the quantity of the additive and not by the type used. Sponge cakes without the addition of insect flour received the highest score from respondents (6.19), while the acceptance for sponge cakes with the insect flour substitution was lower, ranging from 4.63 to 5.56. Increasing the content of the added meal resulted in lower ratings for all parameters assessed by the evaluation team. Table 11 displays the results. The sponge cake supplemented with the 15% insect meal achieved a high level of acceptability on a seven-point scale, irrespective of the insect species used.

Equations were formulated using the component factors that influenced the acceptability for every variant analyzed in the data study to establish which of the examined sensory parameters had the most significant effect on overall acceptability. Table 12 shows the resulting multiple general-acceptability equations.

Preliminary regression analysis indicated that the results of the sensory characteristics can be generalized by omitting the amount of added insect flour. Multiple regression analysis showed that, for sponge cakes with the addition of insect meal, the smell turned out to be insignificant and had no effect on the overall acceptability of the tested sponge cakes, or had only a slight effect. The results obtained demonstrate that the acceptability level was determined by the predictors incorporated in the regression equation, with taste being the most significant factor for overall acceptability, followed by structure and appearance, to a lesser extent. The correlation coefficient R^2^ measured for all tested samples was high at 0.99, thereby highlighting the model’s ability to explain almost 99% of the dependent variable’s variability. The remaining 10% of the variability was attributed to unanalyzed parameters. The regression analysis identified taste as the critical factor in determining the overall acceptability. It was observed that the greater the inclusion of insect meal, the lower the taste rating. The high x1 coefficient, exceeding 0.5 each time (0.73 for *T. molitor*), made the taste the most important element in determining acceptability and the final rating set by the testers.

The current results find partial confirmation in the literature. However, sensory evaluation depends on the type of product and the amount of additive used. Roncolini et al. [47] observed a decline in the consumer acceptability of bread enriched with *T. molitor* flour. However, they did not find any significant impact on overall product acceptability when varying the additive level (5% and 10%). In the authors’ previous study [6], a decrease was observed in the organoleptic quality of sponge cakes regardless of the proportion of mealworm flour incorporated into them. Ruszkowska et al. [11] observed that, in extruded corn snacks, the inclusion of up to 6% cricket flour allowed for the production of sensory-appealing snacks.

## 4. Conclusions

The addition of insect flours had a significant impact on the nutritional properties and consumer acceptance of the obtained sponge cakes. Both the raw materials and sponge cakes exhibited high values of the n-6/n-3 ratio, indicating an imbalance in the lipid fractions; however, with a relatively high oleic acid content. For all the analyzed sponge cakes’ essential amino acids, the AAS exceeded 100%. The range was from 100.15% (lysine in CF30) to 216.43% (for AAA in the TF15 sample). The introduction of edible insects to the biscuit recipe also increased their antioxidant potential, although one should be aware that this type of addition will not be crucial in increasing the antioxidant value of the product as much as plant additives. Sponge cakes supplemented with flour from edible insects are also characterized by an increase in the content of dietary fiber. This increase is not only due to the fiber content of the flours themselves, but also due to the interaction with starch and the formation of resistant starch. Products with the addition of edible insects are generally well-accepted by consumers, and the most important quality factor affecting acceptance is taste. The conducted research indicates that the use of edible insects in the form of flours added to confectionary products may both gain consumer acceptance and contribute to the improvement of the prohealth properties of these products.

## Figures and Tables

**Table 1:** Chemical composition of the raw materials and products (g/100 g).

Samples	Moisture	Protein	Ash	Fat	Dietary Fiber			
					Insoluble Fraction	Including Chitin	Soluble Fraction	Total
WF	14.34 ± 0.02 ^c^	10.54 ± 0.11 ^i^	0.40 ± 0.00 ^j^	1.23 ± 0.01 ^i^	0.43 ± 0.00 ^i^	0.00 ± 0.00 ^j^	1.11 ± 0.05 ^a^	1.53 ± 0.01 ^i^
BF	2.58 ± 0.11 ^h^	44.64 ± 0.13 ^b^	4.71 ± 0.03 ^a^	26.44 ± 0.22 ^b^	12.96 ± 0.04 ^b^	7.33 ± 0.01 ^b^	0.00 ± 0.00 ^i^	12.96 ± 0.04 ^b^
CF	4.50 ± 0.01 ^f^	50.68 ± 0.09 ^a^	4.34 ± 0.07 ^b^	29.01 ± 0.04 ^a^	18.48 ± 0.06 ^a^	9.92 ± 0.01 ^a^	0.24 ± 0.01 ^h^	18.72 ± 0.05 ^a^
TF	3.89 ± 0.07 ^g^	41.22 ± 0.04 ^c^	3.86 ± 0.01 ^c^	14.29 ± 0.06 ^c^	11.56 ± 0.07 ^c^	6.93 ± 0.02 ^c^	0.36 ± 0.02 ^g^	11.92 ± 0.05 ^c^
S	14.04 ± 0.22 ^c^	14.93 ± 0.04 ^h^	0.98 ± 0.01 ^i^	8.24 ± 0.03 ^h^	0.39 ± 0.01 ^i^	0.00 ± 0.00 ^j^	1.02 ± 0.00 ^c^	1.41 ± 0.03 ^j^
BF15	14.07 ± 0.16 ^c^	17.32 ± 0.05 ^f^	1.16 ± 0.01 ^gh^	9.90 ± 0.01 ^f^	1.03 ± 0.03 ^h^	0.33 ± 0.01 ^i^	0.98 ± 0.05 ^dc^	2.01 ± 0.02 ^h^
BF30	17.58 ± 0.40 ^a^	19.47 ± 0.07 ^d^	1.29 ± 0.01 ^f^	11.49 ± 0.06 ^d^	1.44 ± 0.00 ^f^	0.55 ± 0.00 ^f^	0.67 ± 0.01 ^e^	2.11 ± 0.01 ^g^
CF15	10.66 ± 0.11 ^e^	17.45 ± 0.08 ^f^	1.13 ± 0.01 ^h^	9.35 ± 0.30 ^g^	1.40 ± 0.01 ^f^	0.44 ± 0.00 ^g^	1.05 ± 0.01 ^b^	2.44 ± 0.01 ^f^
CF30	12.91 ± 0.04 ^d^	19.37 ± 0.01 ^d^	1.41 ± 0.01 ^d^	10.67 ± 0.13 ^e^	2.06 ± 0.05 ^d^	0.79 ± 0.01 ^d^	0.96 ± 0.01 ^d^	3.01 ± 0.06 ^d^
TF15	11.06 ± 0.12 ^e^	16.50 ± 0.05 ^g^	1.18 ± 0.02 ^g^	9.87 ± 0.01 ^f^	1.21 ± 0.04 ^g^	0.39 ± 0.01 ^h^	0.96 ± 0.01 ^d^	2.17 ± 0.02 ^g^
TF30	15.15 ± 0.44 ^b^	18.67 ± 0.03 ^e^	1.34 ± 0.03 ^e^	10.73 ± 0.16 ^e^	1.92 ± 0.00 ^e^	0.66 ± 0.00 ^e^	0.92 ± 0.01	2.84 ± 0.01 ^e^

Explanatory notes: WF—wheat flour; BF—buffalo worm (*A. diaperinus*) flour; CF—cricket (*A. domesticus*) flour; TF—mealworm (*T. molitor*) flour; S—standard (wheat sponge cake); BF15, BF30—sponge cake with 15 and 30% addition of buffalo worm flour; CF15, CF30—sponge cake with 15 and 30% addition of cricket flour; TF15, TF30—sponge cake with 15 and 30% addition of mealworm flour. Values in the same column marked with different letters are statistically significantly different at *p* < 0.05 ± SD.

**Table 4:** Fatty acid profile of the sponge cakes and insect flour (g/100 g).

Fatty Acids	BF	CF	TF	S	BF15	BF30	CF15	CF30	TF15	TF30
C16:0	27.21 ± 0.02 ^b^	28.90 ± 0.00 ^a^	23.34 ± 0.14 ^g^	24.46 ± 0.07 ^f^	25.25 ± 0.25 ^e^	25.21 ± 0.50 ^e^	26.03 ± 0.07 ^c^	27.13 ± 0.23 ^b^	25.48 ± 0.04 ^e^	25.85 ± 0.07 ^d^
C16:1	0.32 ± 0.01 ^i^	0.69 ± 0.02 ^h^	1.95 ± 0.03 ^g^	3.05 a ± 0.01 ^a^	2.51 ± 0.01 ^d^	2.15 ± 0.04 g	2.46 ± 0.00 ^e^	2.40 ± 0.03 ^f^	2.78 ± 0.01 ^c^	2.90 ± 0.01 ^b^
C17:0	0.49 ± 0.00 ^a^	0.28 ± 0.00 ^b^	0.18 ± 0.01 ^ef^	0.17 ± 0.01 ^f^	0.21 ± 0.00 ^c^	0.25 ± 0.01 ^b^	0.21 ± 0.01 ^c^	0.20 ± 0.01 ^dc^	0.19 ± 0.01 ^ef^	0.19 ± 0.00 ^ef^
C17:1	0.14 ± 0.01 ^a^	0.02 ± 0.03 ^e^	0.10 ± 0.00 ^bc^	0.10 ± 0.01 ^bc^	0.09 ± 0.00 ^bc^	0.11 ± 0.02 ^a^	0.09 ± 0.00 ^bc^	0.07 ± 0.00 ^d^	0.09 ± 0.00 ^bc^	0.10 ± 0.00 ^bc^
C18:0	8.55 ± 0.01 ^d^	11.86 ± 0.04 ^a^	4.68 ± 0.04 ^i^	7.95 ± 0.07 ^h^	8.36 ± 0.08 ^e^	8.13 ± 0.18 ^fg^	8.92 ± 0.02 ^c^	9.56 ± 0.08 ^b^	8.21 ± 0.04 ^ef^	7.98 ± 0.04 ^gh^
C18:1	34.12 ± 0.00 ^f^	25.85 ± 0.00 ^g^	54.87 ± 0.36 ^a^	39.99 ± 0.25 ^d^	40.22 ± 0.24 ^d^	39.71 ± 0.90 ^d^	40.01 ± 0.08 ^d^	36.00 ± 0.25 ^e^	44.98 ± 0.13 ^b^	43.92 ± 0.08 ^c^
C18:2 n-6 cis	24.84 ± 0.02 ^a^	23.36 ± 0.05 ^b^	7.80 ± 0.04 ^i^	18.07 ± 0.12 ^e^	17.24 ± 0.09 ^f^	19.07 ± 0.42 ^c^	17.83 ± 0.03 ^e^	18.50 ± 0.15 ^d^	13.03 ± 0.04 ^h^	14.21 ± 0.02 ^g^
C18:2 n-6 trans	0.10 ± 0.00 ^b^	0.28 ± 0.00 ^a^	0.11 ± 0.01 ^b^	0.02 g ± 0.00 ^g^	0.02 ± 0.00 ^g^	0.05 ± 0.00 ^d^	0.04 ± 0.00 ^e^	0.08 ± 0.01 ^c^	0.03 ± 0.00 ^f^	0.03 ± 0.00 ^f^
C18:3 n-6	0.05 ± 0.00 ^fg^	0.02 ± 0.03 ^g^	0.20 ± 0.00 ^a^	0.13 b ± 0.00 ^b^	0.09 ± 0.02 ^cde^	0.10 ± 0.02 ^bcd^	0.07 ± 0.00 ^ef^	0.08 ± 0.00 ^de^	0.07 ± 0.00 ^ef^	0.11 ± 0.00 ^bc^
C18:3 n-3	1.28 ± 0.00 ^a^	1.21 ± 0.00 ^b^	0.33 ± 0.00 ^g^	1.02 ± 0.00 ^c^	0.78 ± 0.00 ^d^	0.97 ± 0.07 ^c^	0.82 ± 0.01 ^d^	0.82 ± 0.01 ^d^	0.51 ± 0.00 ^f^	0.54 ± 0.00 ^e^
C20:0	0.45 ± 0.01 ^bc^	0.55 ± 0.00 ^b^	0.33 ± 0.00 ^bcd^	0.86 ± 0.08 ^a^	0.91 ± 0.08 ^a^	0.39 ± 0.26 ^bc^	0.30 ± 0.00 ^cd^	0.85 ± 0.18 ^a^	0.13 ± 0.02 ^d^	0.12 ± 0.03 ^d^
C22:6	0.24 ± 0.00 ^f^	0.21 ± 0.02 ^g^	0.19 ± 0.00 ^h^	0.88 ± 0.06 ^a^	0.77 ± 0.05 ^ab^	0.44 ± 0.18 ^cd^	0.47 ± 0.01 ^c^	0.68 ± 0.08 ^b^	0.29 ± 0.05 ^de^	0.27 ± 0.01 ^e^
C20:2	0.08 ± 0.01 ^d^	0.06 ± 0.01 ^e^	0.04 ± 0.01 ^f^	0.44 ± 0.07 ^a^	0.45 ± 0.09 ^a^	0.40 ± 0.04 ^a^	0.33 ± 0.03 ^b^	0.42 ± 0.05 ^a^	0.15 ± 0.02 ^c^	0.16 ± 0.00 ^c^
C21:0	0.76 ± 0.02 ^a^	0.16 ± 0.01 ^d^	0.06 ± 0.0 ^f^	0.34 ± 0.11 ^bc^	0.33 ± 0.09 ^bc^	0.27 ± 0.21 ^bc^	0.21 ± 0.04 ^cd^	0.43 ± 0.20 ^b^	0.09 ± 0.00 ^e^	0.18 ± 0.08 ^cd^
C20:3	0.15 ± 0.00 ^a^	0.01 ± 0.00 ^d^	n.d.	n.d.	0.08 ± 0.05 ^b^	0.15 ± 0.16 ^a^	0.04 ± 0.02 ^c^	n.d.	n.d.	n.d.
C20:3	n.d.	0.02 ± 0.01 ^e^	n.d.	1.20 ± 0.10 ^a^	1.12 ± 0.13 ^ab^	0.99 ± 0.39 ^ab^	0.85 ± 0.04 ^d^	1.12 ± 0.13 ^ab^	0.90 ± 0.00 ^c^	1.04 ± 0.00 ^b^
C20:4	0.01 ± 0.00 ^c^	0.15 ± 0.05 ^a^	n.d.	0.09 ± 0.09 ^ba^	0.20 ± 0.12 ^a^	0.23 ± 0.09 ^a^	0.10 ± 0.12 ^ba^	n.d.	n.d.	n.d.
C20:1	0.05 ± 0.00 ^d^	0.10 ± 0.00 ^b^	0.17 ± 0.01 ^a^	n.d.	n.d.	0.17 ± 0.08 ^a^	0.04 ± 0.02 ^d^	0.04 ± 0.00 ^d^	0.08 ± 0.01 ^c^	0.07 ± 0.01 ^c^
C22:0	0.09 ± 0.00 ^b^	0.11 ± 0.00 ^a^	0.04 c ± 0.00 ^c^	0.04 ± 0.00 ^c^	0.04 ± 0.00 ^c^	0.07 ± 0.03 ^b^	0.04 ± 0.00 ^c^	0.04 ± 0.00 ^c^	0.03 ± 0.00 ^d^	0.05 ± 0.03 ^cd^
C22:1	0.01 ± 0.00 ^c^	1.01 ± 0.02 ^a^	0.02 ± 0.01 ^c^	0.02 ± 0.00 ^c^	n.d.	0.02 ± 0.01 ^c^	0.01 ± 0.00 ^c^	0.08 ± 0.00 ^b^	n.d.	n.d.
C22:2	0.01 ± 0.00 ^e^	1.00 ± 0.01 ^a^	0.02 ± 0.01 ^d^	0.02 ± 0.00 ^d^	0.05 ± 0.02 ^c^	0.08 ± 0.01 ^b^	0.04 ± 0.00 ^c^	0.07 ± 0.00 ^b^	0.04 ± 0.04 ^c^	0.01 ± 0.00 ^e^
C23:0	0.02 ± 0.01 ^d^	0.48 ± 0.05 ^b^	0.05 ± 0.03 ^cd^	0.08 ± 0.00 ^c^	0.02 ± 0.01 ^d^	0.02 ± 0.01 ^d^	0.03 ± 0.00 ^d^	0.03 ± 0.00 ^cd^	0.65 ± 0.00 ^a^	0.02 ± 0.01 ^d^
C24:0	0.05 ± 0.00 ^c^	0.22 ± 0.00 ^a^	0.10 ± 0.01 ^b^	0.05 ± 0.01 ^c^	0.04 ± 0.03 ^c^	0.05 ± 0.03 ^c^	0.02 ± 0.01 ^d^	0.02 ± 0.00 ^d^	0.02 ± 0.01 ^d^	0.03 ± 0.02 ^c^
C20:5	n.d.	0.08 ± 0.00 ^d^	n.d.	0.42 ± 0.00 ^a^	0.41 ± 0.05 ^a^	0.29 ± 0.02 ^c^	0.31 ± 0.01 ^c^	0.37 ± 0.00 ^b^	0.32 ± 0.00 ^c^	0.36 ± 0.00 ^b^
C24:1	0.01 ± 0.00 ^d^	2.36 ± 0.07 ^a^	0.17 ± 0.01 ^b^	0.01 ± 0.00 ^d^	0.10 ± 0.01 ^c^	0.01 ± 0.01 ^d^	0.09 ± 0.01 ^c^	0.18 ± 0.00 ^b^	0.04 ± 0.04 ^cd^	0.05 ± 0.05 ^cd^
∑ SFA	37.62 ± 0.01 ^c^	42.54 ± 0.08 ^a^	28.80 ± 0.28 ^h^	33.94 ± 0.03 ^g^	35.16 ± 0.08 ^d^	34.38 ± 0.07 ^f^	35.75 ± 0.04 ^d^	38.27 ± 0.05 ^b^	34.80 ± 0.04 ^e^	34.41 ± 0.04 ^f^
∑ MUFA	34.64 ± 0.02 ^g^	30.03 ± 0.04 ^h^	57.28 ± 0.31 ^a^	43.16 ± 0.24 ^d^	42.92 ± 0.27 ^d^	42.16 ± 0.07 ^e^	42.70 ± 0.13 ^d^	38.78 ± 0.27 ^f^	47.97 ± 0.10 ^b^	47.03 ± 0.03 ^c^
∑ PUFA	26.76 ± 0.03 ^a^	26.42 ± 0.04 ^a^	8.69 ± 0.03 ^f^	22.27 ± 0.21 ^b^	21.20 ± 0.45 ^c^	22.76 ± 0.94 ^b^	20.89 ± 0.17 ^c^	22.13 ± 0.23 ^b^	15.34 ± 0.06 ^e^	16.73 ± 0.01 ^d^
∑PUFA/∑SFA	0.69 ± 0.00 ^a^	0.61 ± 0.00 ^c^	0.30 ± 0.00 ^g^	0.65 ± 0.01 ^b^	0.59 ± 0.02 ^d^	0.65 ± 0.03 ^b^	0.57 ± 0.01 ^d^	0.57 ± 0.01 ^d^	0.42 ± 0.00 ^f^	0.46 ± 0.00 ^e^
n6	25.24 ± 0.03 ^a^	24.90 ± 0.03 ^b^	8.17 ± 0.03 ^j^	18.77 ± 0.03 ^e^	18.12 ± 0.22 ^g^	20.08 ± 0.28 ^c^	18.45 ± 0.13 ^f^	19.15 ± 0.01 ^d^	13.32 ± 0.01 ^i^	14.52 ± 0.02 ^h^
n3	1.52 ± 0.00 ^f^	1.53 ± 0.01 ^f^	0.52 ± 0.00 ^g^	3.51 ± 0.18 ^a^	3.07 ± 0.22 ^ab^	2.68 ± 0.66 ^bc^	2.43 ± 0.04 ^c^	2.98 ± 0.22 ^ab^	2.02 ± 0.04 ^e^	2.21 ± 0.01 ^d^
n6/n3	16.56 ± 0.03 ^a^	16.29 ± 0.08 ^b^	15.83 ± 0.10 ^c^	5.36 ± 0.26 ^f^	5.91 ± 0.36 ^f^	7.72 ± 1.80 ^d^	7.58 ± 0.06 ^d^	6.43 ± 0.47 ^e^	6.60 ± 0.13 ^e^	6.58 ± 0.05 ^e^

Explanatory notes: meaning of the symbols as in Table 1; n.d.—not detected. Values in the same row marked with different letters are statistically significantly different at *p* < 0.05 ± SD.

**Table 5:** Color parameters of the sponge cakes.

Color Parameters	L*	a*	b*	∆E
S	62.31 ± 2.38 ^a^	14.25 ± 0.93 ^a^	34.63 ± 0.70 ^a^	-
BF15	60.28 ± 1.95 ^b^	12.66 ± 0.98 ^b^	32.53 ± 0.61 ^b^	3.80 ± 0.85 ^c^
BF30	57.60 ± 1.34 ^c^	12.17 ± 0.85 ^bc^	31.42 ± 0.52 ^c^	5.86 ± 1.02 ^b^
CF15	57.46 ± 1.14 ^c^	12.37 ± 0.83 ^b^	31.41 ± 0.27 ^c^	5.91 ± 0.71 ^b^
CF30	56.51 ± 1.04 ^c^	11.56 ± 0.43 ^c^	29.14 ± 0.26 ^e^	7.26 ± 0.62 ^a^
TF15	61.04 ± 0.95 ^ab^	11.54 ± 0.44 ^c^	31.09 ± 0.22 ^cd^	4.12 ± 0.24 ^c^
TF30	57.57 ± 1.77 ^c^	12.05 ± 1.18 ^bc^	30.71 ± 0.25 ^d^	6.19 ± 1.02 ^b^

Explanatory notes: meaning of the symbols as in Table 1. Values in the same column marked with different letters are statistically significantly different at *p* < 0.05 ± SD.

**Table 6:** Total polyphenol and flavonoid content in the sponge cakes.

Sample	TPC (mg Catechin/100 g d.m.)	Flavonoids (mg Rutin/100 g d.m.)
S	43.58 ± 0.00 ^g^	13.95 ± 0.97 ^c^
BF15	99.38 ± 4.64 ^e^	14.37 ± 0.60 ^c^
BF30	134.0 ± 2.31 ^a^	19.44 ± 0.60 ^b^
CF15	102.12 ± 0.77 ^d^	11.42 ± 1.50 ^c^
CF30	155.73 ± 3.86 ^b^	19.86 ± 1.19 ^b^
TF15	82.42 ± 2.32 ^f^	19.87 ± 0.0 ^b^
TF30	120.56 ± 0.77 ^c^	34.65 ± 0.60 ^a^

Explanatory notes: meaning of the symbols as in Table 1. Values in the same column marked with different letters are statistically significantly different at *p* < 0.05 ± SD.

**Table 7:** Profile of the phenolic compounds of the sponge cakes.

Compounds	S	BF15	BF30	CF15	CF30	TF15	TF30
(mg/g)							
Gallic acid	0.77 ± 0.019 ^f^	16.83 ± 0.422 ^b^	31.72 ± 0.795 ^a^	6.52 ± 0.164 ^d^	7.01 ± 0.176 ^c^	4.00 ± 0.100 ^e^	16.05 ± 0.403 ^b^
Vanillic acid	0.00 ± 0.000 ^d^	10.90 ± 0.273 ^c^	16.35 ± 0.410 ^b^	10.90 ± 0.199 ^c^	16.25 ± 0.310 ^b^	11.00 ± 0.547 ^c^	22.00 ± 0.281 ^a^
Protocatechuic acid	0.00 ± 0.000 ^f^	68.44 ± 1.634 ^d^	136.89 ± 3.433 ^b^	50.00 ± 0.989 ^e^	109.00 ± 1.119 ^c^	68.45 ± 1.716 ^d^	239.56 ± 6.008 ^a^
Syringic acid	0.00 ± 0.000 ^c^	0.00 ± 0.000 ^c^	191.77 ± 4.809 ^a^	0.00 ± 0.000 ^c^	0.00 ± 0.000 ^c^	0.00 ± 0.000 ^c^	0.98 ± 0.024 ^b^
Protocatechuic aldehyde	6.61 ± 1.751 ^g^	69.81 ± 1.751 ^d^	125.60 ± 3.150 ^a^	39.30 ± 0.986 ^f^	54.33 ± 1.362 ^e^	71.45 ± 1.792 ^c^	100.35 ± 2.517 ^b^
Ellagic acid	0.00 ± 0.000 ^e^	1.08 ± 0.016 ^d^	2.15 ± 0.047 ^c^	23.18 ± 0.581 ^b^	52.96 ± 1.328 ^a^	2.15 ± 0.054 ^c^	1.08 ± 0.027 ^d^
Total HBA	7.37 ± 4.189 ^g^	167.06 ± 12.651 ^d^	504.48 ± 12.651 ^a^	129.90 ± 2.004 ^f^	239.65 ± 3.276 ^c^	157.05 ± 4.209 ^e^	380.02 ± 9.259 ^b^
Caffeic acid	4.00 ± 0.000 ^c^	0.00 ± 0.000 ^d^	0.00 ± 0.000 ^d^	6.53 ± 0.647 ^b^	24.44 ± 2.421 ^a^	0.00 ± 0.000 ^d^	0.00 ± 0.000 ^d^
Ferulic acid	7.76 ± 0.055 ^d^	5.81 ± 0.575 ^f^	8.37 ± 0.829 ^c^	24.01 ± 2.378 ^b^	61.55 ± 6.096 ^a^	6.89 ± 0.682 ^e^	8.79 ± 0.870 ^c^
*p*-Coumaric acid	3.00 ± 0.001 ^a^	0.00 ± 0.000 ^b^	0.00 ± 0.000 ^b^	0.00 ± 0.000 ^b^	0.00 ± 0.000 ^b^	0.00 ± 0.000 ^b^	0.00 ± 0.000 ^b^
Sinapic acid	0.00 ± 0.000 ^d^	0.00 ± 0.000 ^d^	0.00 ± 0.000 ^d^	24.46 ± 2.422 ^b^	54.65 ± 5.412 ^a^	0.00 ± 0.000 ^d^	10.14 ± 1.004 ^c^
Total HCA	14.76 ± 0.055 ^d^	5.81 ± 0.574 ^g^	8.37 ± 0.816 ^e^	54.99 ± 5.447 ^b^	140.63 ± 13.929 ^a^	6.89 ± 0.678 ^f^	18.92 ± 1.874 ^c^

Explanatory notes: meaning of the symbols as in Table 1. Values in the same row marked with different letters are statistically significantly different at *p* < 0.05 ± SD.

**Table 8:** Antioxidant activity of the sponge cakes.

Samples	DPPH (mg Tx/g)	ABTS (mg Tx/g)	FRAP (mg Fe^2+^/g)	Iron Reduction
S	4.95 ± 0.30 ^e^	5.98 ± 0.13 ^f^	22.2 ± 0.12 ^g^	884.96 ± 0.89 ^a^
BF15	7.46 ± 0.16 ^c^	9.02 ± 0.00 ^d^	33.3 ± 0.95 ^e^	233.45 ± 1.03 ^d^
BF30	10.32 ± 0.38 ^b^	12.46 ± 0.21 ^b^	45.33 ± 0.50 ^b^	179.25 ± 1.32 ^e^
CF15	6.63 ± 0.11 ^d^	7.99 ± 0.11 ^e^	29.58 ± 0.19 ^f^	233.06 ± 0.14 ^d^
CF30	13.2 ± 0.00 ^a^	15.93 ± 0.25 ^a^	58.3 ± 0.66 ^a^	138.89 ± 1.67 ^f^
TF15	7.36 ± 0.11 ^c^	9.58 ± 0.67 ^d^	35.15 ± 0.28 ^d^	333.50 ± 0.00 ^b^
TF30	9.94 ± 0.00 ^b^	11.13 ± 0.10 ^c^	40.7 ± 0.68 ^c^	312.33 ± 0.65 ^c^

Explanatory notes: meaning of the symbols as in Table 1. Values in the same column marked with different letters are statistically significantly different at *p* < 0.05 ± SD.

**Table 9:** The content of volatile compounds (%) in the sponge cakes.

Volatile Compounds (VC)	VC Groups	S	BF15	BF30	CF15	CF30	TF15	TF30
Acetic acid	Acids	n.d.	n.d.	n.d.	n.d.	0.56 ± 0.06	n.d.	n.d.
Butanoic acid		0.31 ± 0.03	0.46 ± 0.05	0.48 ± 0.05	0.62 ± 0.06	1.07 ± 0.11	1.13 ± 0.11	1.73 ± 0.17
3-Methylobutanoic acid		n.d.	n.d.	n.d.	n.d.	n.d.	n.d.	n.d.
Hexanoic acid		n.d.	n.d.	n.d.	0.29 ± 0.03	0.29 ± 0.03	n.d.	n.d.
Myristic acid		0.27 ± 0.03	0.34 ± 0.03	0.00	0.37 ± 0.04	0.25 ± 0.02	0.19 ± 0.02	0.14 ± 0.01
2-propanol	Alcohols	13.34 ± 1.33	16.70 ± 0.65	16.38 ± 1.63	14.93 ± 1.49	17.80 ± 0.78	11.92 ± 1.18	10.17 ± 1.02
1-Hexanol		0.25 ± 0.02	0.21 ± 0.02	n.d.	0.20 ± 0.02	0.47 ± 0.05	0.20 ± 0.02	0.11 ± 0.01
2-Phenylethanol		0.87 ± 0.09	0.57 ± 0.06	0.79 ± 0.08	0.47 ± 0.05	0.39 ± 0.04	0.45 ± 0.04	0.61 ± 0.06
Acetaldehyde	Aldehydes	5.12 ± 0.51	4.28 ± 0.42	4.59 ± 0.45	4.34 ± 0.43	3.91 ± 0.39	3.17 ± 0.31	2.62 ± 0.26
2-Methylpropanal		1.36 ± 0.14	1.17 v0.12	1.54 ± 0.15	1.03 ± 0.10	1.01 ± 0.10	0.69 ± 0.07	0.63 ± 0.06
Butanal		17.03 ± 0.70	24.08 ± 1.38	24.94 ± 1.47	18.59 ± 0.86	15.43 ± 1.55	19.10 ± 0.89	14.52 ± 1.46
3-Methylbutanal		24.53 ± 1.44	16.24 ± 1.06	15.07 ± 1.49	18.82 ± 0.88	14.54 ± 1.46	16.42 ± 0.63	12.12 ± 1.02
Hexanal		1.46 ± 0.15	2.59 ± 0.26	2.24 ± 0.22	3.23 ± 0.32	4.71 ± 0.47	4.28 ± 0.42	6.18 ± 0.62
Furfural		2.26 ± 0.22	4.53 ± 0.45	3.62 ± 0.36	7.60 ± 0.76	15.65 ± 1.57	15.01 ± 1.49	25.03 ± 2.52
Heptanal		0.12 ± 0.01	0.40 ± 0.04	0.32 ± 0.03	0.46 ± 0.05	0.88 ± 0.09	1.07 ± 0.11	1.36 ± 0.14
2.4-Heptadienal		0.27 ± 0.03	0.50 ± 0.05	00.28 ± 0.03	1.57 ± 0.00	1.72 ± 0.17	0.18 ± 0.02	1.63 ± 0.16
Benzaldehyde		n.d.	n.d.	n.d.	n.d.	0.39 ± 0.04	n.d.	n.d.
Dodecanal		n.d.	n.d.	n.d.	0.25 ± n.d.	0.13 ± 0.00	n.d.	n.d.
Ethyl Acetate	Esters	3.67 ± 0.37	3.44 ± 0.34	4.06 ± 0.40	3.61 ± 0.36	3.16 ± 0.32	3.41 ± 0.34	2.87 ± 0.29
Ethyl hexanoate		n.d.	n.d.	n.d.	n.d.	0.80 ± n.d.	n.d.	n.d.
Phenylethyl acetate		0.24 ± 0.02	0.09 ± 0.01	0.00	0.58 ± 0.06	0.00	0.11 ± 0.01	0.05 ± 0.01
Pentyl octanoate		n.d.	n.d.	n.d.	n.d.	n.d.	n.d.	n.d.
Benzyl benzoate		0.71 ± 0.07	0.70 ± 0.07	0.74 ± 0.07	0.00	0.00	0.35 ± 0.03	0.31 ± 0.03
Benzyl phenyl acetate		0.55 ± 0.05	0.32 ± 0.03	0.00	0.30 ± 0.03	0.20 ± 0.02	0.23 ± 0.02	0.14 ± 0.01
Myristicin		n.d.	n.d.	n.d.	n.d.	n.d.	n.d.	n.d.
Butan-2-one	Ketones	1.99 ± 0.20	2.32 ± 0.23	2.03 ± 0.23	2.01 ± 0.20	1.56 ± 0.16	1.63 ± 0.16	0.95 ± 0.10
2.3-Pentanedione		20.25 ± 1.02	18.23 ± 0.81	18.35 ± 0.82	16.09 ± 0.61	11.63 ± 1.17	17.40 ± 0.72	13.32 ± 1.33
gamma-Nonalactone	Lactones	0.22 ± 0.02	n.d.	n.d.	n.d.	n.d.	n.d.	n.d.
delta-Nonalacton		0.58 ± 0.06	0.21 ± 0.02	0.30 ± 0.03	00.23 ± 0.02	0.00	0.36 ± 0.04	0.41 ± 0.04
delta-Decalactone		n.d.	n.d.	n.d.	n.d.	n.d.	n.d.	n.d.
Eugenol	Phenols	0.30 ± 0.03	0.41 ± 0.04	0.42 ± 0.04	0.48 ± 0.05	0.23 ± 0.02	0.64 ± 0.06	0.69 ± 0.07
E-nerolidol		0.17 ± 0.02	0.07 ± 0.01	0.27 ± 0.03	0.14 ± 0.01	0.05 ± 0.01	0.19 ± 0.02	0.07 ± 0.01
Guaiol		0.35 ± 0.03	0.41 ± 0.04	0.49 ± 0.05	0.41 ± 0.04	0.27 ± 0.03	0.31 ± 0.03	0.24 ± 0.02
Maltol	Pyranones	0.23 ± 0.02	n.d.	1.17 ± 0.12	n.d.	n.d.	n.d.	n.d.
2.5-Dimethylpyrazine		1.41 ± 0.14	0.83 ± 0.08	0.76 ± 0.08	0.68 ± 0.06	0.48 ± 0.05	0.42 ± 0.04	0.24 ± 0.02
2.3-Dimethylpyrazine		0.37 ± 0.04	0.24 ± 0.02	n.d.	0.58 ± 0.16	0.00	0.21 ± 0.02	0.54 ± 0.05
Trimethylpyrazine		0.52 ± 0.05	n.d.	0.04 ± 0.00	1.01 ± 0.10	1.20 ± 0.12	n.d.	1.70 ± 0.17
Tetramethylpyrazine		1.27 ± 0.13	0.68 ± 0.07	0.74 ± 0.07	1.07 ± 0.11	1.20 ± 0.12	0.91 ± 0.09	1.70 ± 0.17

Explanatory notes: meaning of the symbols as in Table 1; n.d.—not detected. The results revealed that the addition of insect flours significantly influenced the aroma profile of the obtained sponge cakes.

**Table 10:** Thermal parameters of the obtained sponge cakes depending on the type and concentration of insect flour.

Sample	To	Tp	Tc	ΔT	ΔH
	(°C)				(J g^−1^)
S	79.51 ± 0.96	124.11 ± 1.45	162.16 ± 1.69	82.65 ± 1.21	188.74 ± 1.32
BF15	51.83 ± 0.78	121.93 ± 1.05	155.38 ± 0.93	103.55 ± 1.23	247.15 ± 0.96
BF30	51.34 ± 0.68	130.62 ± 0.68	153.90 ± 1.08	102.56 ± 0.57	254.79 ± 1.85
CF15	51.48 ± 0.63	107.58 ± 1.24	156.15 ± 1.04	104.67 ± 0.79	223.30 ± 1.04
CF30	51.53 ± 0.76	121.02 ± 1.72	154.79 ± 0.79	103.26 ± 1.45	243.40 ± 1.21
TF15	45.32 ± 1.05	107.42 ± 0.83	153.95 ± 0.78	108.63 ± 0.73	289.12 ± 0.72
TF30	47.15 ± 0.89	106.67 ± 1.06	152.72 ± 1.23	105,57 ± 0.94	274.75 ± 1.53

Explanatory notes: meaning of the symbols as in Table 1. To, onset temperature; Tp, peak temperature; Tc, conclusion temperature; ∆T, temperature range = (Tc − To); ∆H, enthalpy expressed in J g^−1^ sample.

**Table 11:** The results of the consumer organoleptic evaluation of the sponge cakes.

Sample	Appearance	Odor	Structure	Taste	Acceptance
S	6.43 ± 0.61 ^a^	6.30 ± 0.89 ^a^	5.97 ± 1.10 ^a^	5.86 ± 1.26 ^a^	6.19 ± 0.96 ^a^
BF15	5.81 ± 1.15 ^ab^	5.84 ± 1.30 ^ab^	5.16 ± 1.61 ^b^	5.25 ± 1.51 ^ab^	5.47 ± 1.26 ^ab^
BF30	5.65 ± 1.40 ^b^	5.56 ± 1.41 ^b^	5.02 ± 1.45 ^b^	4.80 ± 1.83 ^b^	5.09 ± 1.54 ^bc^
CF15	6.32 ± 1.18 ^a^	5.82 ± 1.42 ^ab^	5.34 ± 1.72 ^ab^	5.20 ± 1.86 ^ab^	5.61 ± 1.71 ^ab^
CF30	4.92 ± 1.71 ^c^	5.31 ± 1.64 ^b^	4.66 ± 1.57 ^b^	4.38 ± 1.90 ^b^	4.63 ± 1.62 ^c^
TF15	6.15 ± 1.26 ^ab^	5.94 ± 1.49 ^ab^	5.34 ± 1.72 ^ab^	5.13 ± 1.80 ^ab^	5.56 ± 1.38 ^ab^
TF30	5.81 ± 1.34 ^ab^	5.34 ± 1.52 ^b^	5.28 ± 1.58 ^ab^	4.88 ± 1.65 ^b^	5.03 ± 1.58 ^bc^

Explanatory notes: meaning of the symbols as in Table 1. Values in the same column marked with different letters are statistically significantly different at *p* < 0.05 ± SD.

**Table 12:** Multiple equations of the overall sponge cake acceptability.

Type of Additive	Regression Equation	R^2^	F
S	y = 0.5 x1 + 0.18 x2 + 0.02 x3 + 0.3 x4	0.99	42.934
BF (in general 15% + 30%)	y = 0.51 x1 + 0.24 x2 + 0.07 x3 + 0.18 x4	0.99	61.767
CF (in general 15% + 30%)	y = 0.5 x1 + 0.28 x2 + 0.02 x3 + 0.2 x4	0.99	56.977
TF (in general 15% + 30%)	y = 0.73 x1 + 0.11 x2 − 0.1 x3 + 0.26 x4	0.99	60.920

R^2^—determination coefficient; F—statistic value; y—preference; x1—taste; x2—structure; x3—odor; x4—appearance.

## Data Availability

Not applicable.

## Abbreviation

WF—wheat flour; BF—buffalo worm flour; CF—cricket flour; TF—mealworm flour; S—standard sponge cake; BF15, BF30—sponge cake with 15 and 30% addition of buffalo worm flour; CF15, CF30—sponge cake with 15 and 30% addition of cricket flour; TF15, TF30—sponge cake with 15 and 30% addition of mealworm flour.
